# Repetitive Transcranial Magnetic Stimulation (rTMS) Improves Cognitive Impairment and Intestinal Microecological Dysfunction Induced by High-Fat Diet in Rats

**DOI:** 10.34133/research.0384

**Published:** 2024-05-31

**Authors:** Kangyu Jin, Bing Chen, Shengyi Han, Jingyi Dong, Shangping Cheng, Bin Qin, Jing Lu

**Affiliations:** ^1^ Department of Psychiatry, the First Affiliated Hospital, Zhejiang University School of Medicine, Hangzhou 310003, China.; ^2^ The Key Laboratory of Mental Disorder Management in Zhejiang Province, Hangzhou 310003, China.; ^3^ State Key Laboratory for Diagnosis and Treatment of Infectious Diseases, The First Affiliated Hospital, Zhejiang University School of Medicine, Hangzhou City 310003, China.; ^4^ School of Life Sciences, Zhejiang Chinese Medical University, Hangzhou, China.

## Abstract

Consuming a high-fat diet (HFD) is widely recognized to cause obesity and result in chronic brain inflammation that impairs cognitive function. Repetitive transcranial magnetic stimulation (rTMS) has shown effectiveness in both weight loss and cognitive improvement, although the exact mechanism is still unknown. Our study examined the effects of rTMS on the brain and intestinal microecological dysfunction. rTMS successfully reduced cognitive decline caused by an HFD in behavioral assessments involving the Y maze and novel object recognition. This was accompanied by an increase in the number of new neurons and the transcription level of genes related to synaptic plasticity (spindlin 1, synaptophysin, and postsynaptic protein-95) in the hippocampus. It was reached that rTMS decreased the release of high mobility group box 1, activation of microglia, and inflammation in the brains of HFD rats. rTMS also reduced hypothalamic hypocretin levels and improved peripheral blood lipid metabolism. In addition, rTMS recovered the HFD-induced gut microbiome imbalances, metabolic disorders, and, in particular, reduced levels of the microvirus. Our research emphasized that rTMS enhanced cognitive abilities, resulting in positive impacts on brain inflammation, neurodegeneration, and the microbiota in the gut, indicating the potential connection between the brain and gut, proposing that rTMS could be a new approach to addressing cognitive deficits linked to obesity.

## Introduction

Consuming a diet high in fat can result in obesity and a persistent low-level inflammation in the brain, specifically in areas like the hypothalamus and hippocampus [1]. This suggests a strong connection between the mechanisms that cause obesity and mood disorders affecting both the body and the brain [2]. Elevations in body mass index may cause metabolic alterations in the brain, potentially caused by inflammatory indicators, resulting in reduced inhibitory regulation [3]. Maayan et al. [3] have linked cognitive decline to the existence of inflammatory markers in individuals who are obese. Prior research indicated that neuroinflammation caused by obesity was linked to the breakdown of the blood–brain barrier, triggering inflammatory responses in the hippocampus and potentially increasing oxidative stress, resulting in cognitive impairment [4].

Studies have shown that repetitive transcranial magnetic stimulation (rTMS) is effective in treating neurological and psychiatric conditions in adult patients [5]. Recent research has shown that applying rTMS to the dorsolateral prefrontal cortex can decrease food consumption and body weight while also enhancing connectivity in the right frontoparietal network when compared to sham stimulation [6]. Other techniques such as transcranial direct current stimulation could also reduce food consumption and induce weight loss [7].

rTMS revealed an improvement in cognitive functions in clinical and preclinical. rTMS has shown promise as a potential therapy for mild cognitive impairment and Alzheimer’s disease in clinical [8]. In the preclinical setting, treatment with 10-Hz rTMS reduced neuronal cell death and enhanced cognitive abilities [9]. Low-intensity rTMS at 10 Hz, rather than high frequency at 40 Hz, can have a notable impact on cognitive function based on the intensity and frequency used [10]. In other studies, rTMS has been shown to improve memory by enhancing long-term potentiation and increasing spine density in the dentate gyrus (DG) region of the hippocampus in mice [11]. It has also been found to protect against the decline of long-term memories in 5xFAD mice, reduce amyloid-β deposits, suppress microglia and astrocyte activation, and prevent the decrease in neuronal activity, as evidenced by elevated c-FOS expression [12]. Transcranial magneto-acoustic stimulation has also been reported to alleviate neuroinflammation and synaptic plasticity damage in 5xFAD mice [13].

Several studies have demonstrated the pivotal role of microglia in neuropsychiatric disorders. rTMS has been found to significantly attenuate microglial activation, suppress elevated levels of interleukin-6 (IL-6), IL-1β, and tumor necrosis factor-α (TNF-α) induced by stress, and induce a shift in microglial polarization from the proinflammatory M1 phenotype to the anti-inflammatory M2 phenotype within the hippocampus and prefrontal cortex [14]. High mobility group box 1 (HMGB1), a nuclear protein with high electrophoretic mobility, is ubiquitously expressed across cell types and plays a crucial role in modulating inflammation and immune response upon release from injured cells [15]. Hypocretin neurons project extensively to key regions such as the prefrontal cortex, hippocampus, thalamus, and hypothalamus that govern various important functions. In recent years, emerging evidence has linked hypocretin neurons not only to appetite regulation and energy homeostasis but also to neuroinflammation [16]. Furthermore, our previous investigations have suggested an association between hypocretin and cognitive function [17].

This study explores the impact of rTMS on obesity and cognitive decline induced by a high-fat diet (HFD). We found that it reduces the release of HMGB1 from neurons, inhibits the activation of microglia, enhances the neuroplasticity of hippocampus, reduces the production of hypocretins, and alleviates dyslipidemia. Notably, rTMS maintains intestinal microbial and metabolic homeostasis. This work presents a strategy targeting obesity and cognitive impairment caused by high-nutrient diets.

## Results

### Effects of HFD on body weight and behavior in rats

**Fig. 1.**
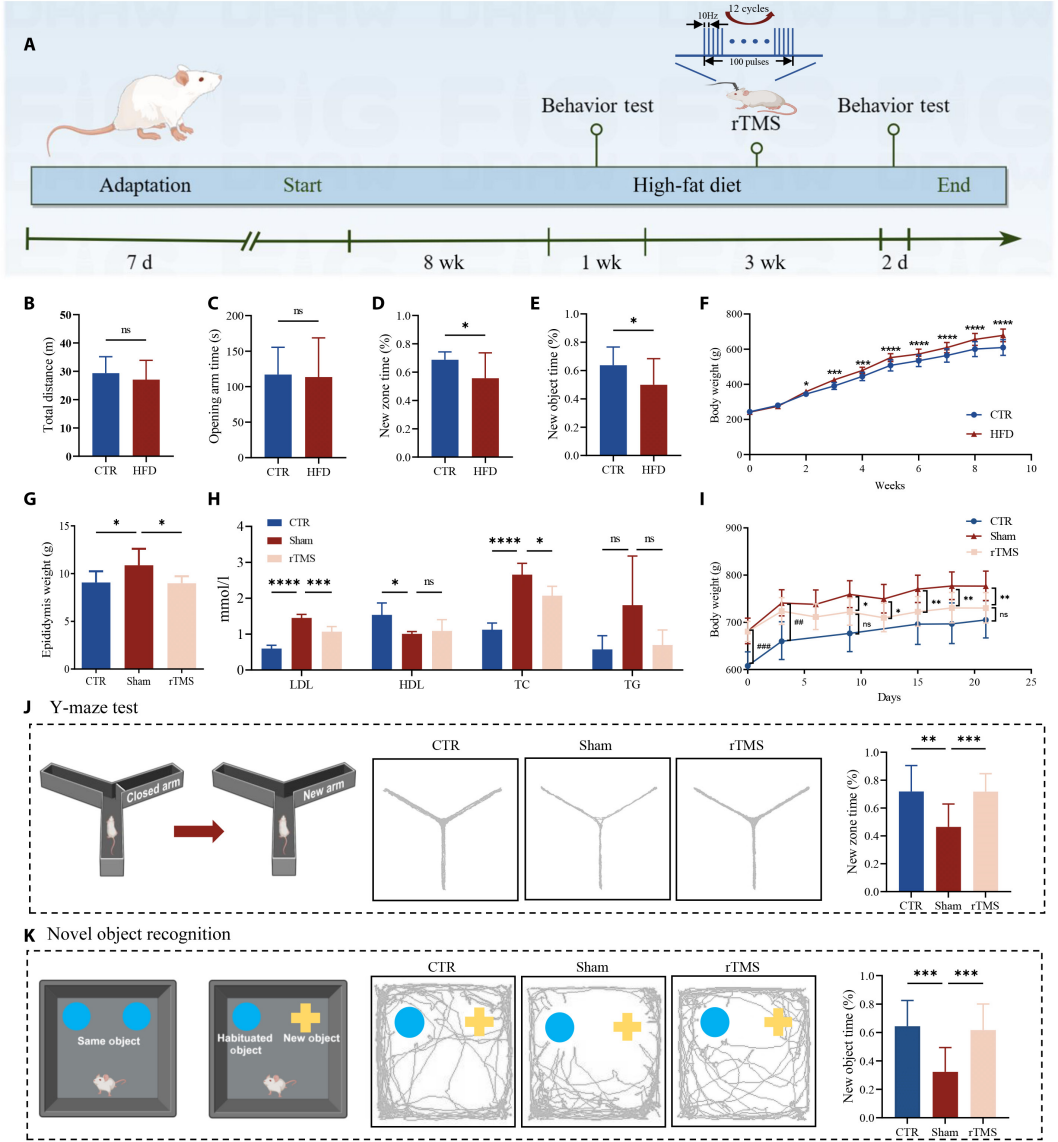
rTMS alleviated HFD-induced cognitive impairment. (A) Timeline of the experiment. (B) The total distance traveled by rats in the open field after an 8-week HFD (*n* = 12 CTR and *n* = 24 HFD). (C) Time spent in the opening arm of the EPM in rats following an 8-week HFD (*n* = 12 CTR and *n* = 24 HFD). (D) Proportion of time spent exploring new zone in Y-maze test after an 8-week HFD (*n* = 12 CTR and *n* = 24 HFD). (E) Proportion of time spent exploring new objects in NOR after an 8-week HFD (*n* = 10 CTR and *n* = 22 HFD). (F) Body weight of rats during an 8-week HFD (*n* = 12 CTR and *n* = 24 HFD). (G) Epididymis weight of rats in different groups (*n* = 6). (H) Plasma LDL, HDL, TC, and TG levels after treatment with rTMS (*n* = 4). (I) Body weight of rats during rTMS treatment (*n* = 12). (J) Proportion of time spent exploring new arm in Y-maze test and representative traces after 3-week rTMS treatment (*n* = 11 CTR, *n* = 12 sham, and *n* = 12 rTMS). (K) Proportion of time spent exploring new objects in NOR and representative traces after 3-week rTMS treatment (*n* = 12). Data are presented as means ± SD. Not significant (ns) *P* ≥ 0.05, *P < 0.05, **P < 0.01, ***P < 0.001, and ****P < 0.0001.

The impact of an HFD on the weight and behavior of Sprague–Dawley (SD) rats was studied by dividing them into 2 groups: control group and HFD group. Over a period of 2 weeks to 2 months, the rats in the HFD group showed a significantly higher weight compared to the control group (Fig. 1F). Behavioral tests were performed on groups to investigate the impact of an HFD on anxiety-related behavior and cognitive abilities. The intermediate time and total distance did not vary between the 2 groups during the open field test (OFT) (Fig. 1B). During the elevated plus maze (EPM) test, the open arm time did not show a significant discrepancy between the 2 groups Fig. 1C). These data showed that 2 months of an HFD did not cause significant anxiety-like behavior and impaired motor ability in the rats. Spatial memory was assessed using the Y-maze. During the Y-maze experiment, rats in the HFD group showed similar times spent in the old and new arms, suggesting a decline in spatial memory (Fig. 1D). The novel object recognition test (NOR) enables rats to engage in learning and memory assessments while freely active, providing a closer representation of human learning and memory patterns. The same HFD group of rats did not have a clear preference for new and old objects (Fig. 1E), indicating that learning and memory ability were impaired.

### The impact of rTMS on cognitive abilities and weight in rats fed an HFD

To study the healing impact of 10-Hz rTMS on rats fed an HFD, we randomly split the HFD group into 2 groups: one receiving an HFD and sham-rTMS (sham) and the other receiving an HFD and true rTMS (rTMS). rTMS therapy effectively decreased the weight increase induced by an HFD (Fig. 1I). During the Y-maze assessment, the amount of time spent in the new arm was notably reduced in the sham group compared to the control (CTR) and rTMS groups, as shown in Fig. 1J. Likewise, during the NOR, the amount of time spent with new objects in the sham group was notably less than in the CTR and rTMS groups (Fig. 1K). The findings indicated that rTMS partially reversed the cognitive deficits induced by an HFD. Concurrently, the mass of epididymal white adipose tissue in the rTMS cohort was notably reduced compared to the sham cohort (Fig. 1G). Furthermore, we assessed 4 markers that are strongly linked to obesity: total cholesterol (TC), low-density lipoprotein (LDL), high-density lipoprotein (HDL), and triglyceride (TG). In comparison to the control group, the sham group showed a significant increase in LDL and TC levels, a significant decrease in HDL levels, and an increase in TG levels with no significant difference observed. rTMS treatment significantly reduced LDL and TC levels (Fig. 1H).

### Impact of rTMS on neural plasticity in rats fed an HFD

**Fig. 2.**
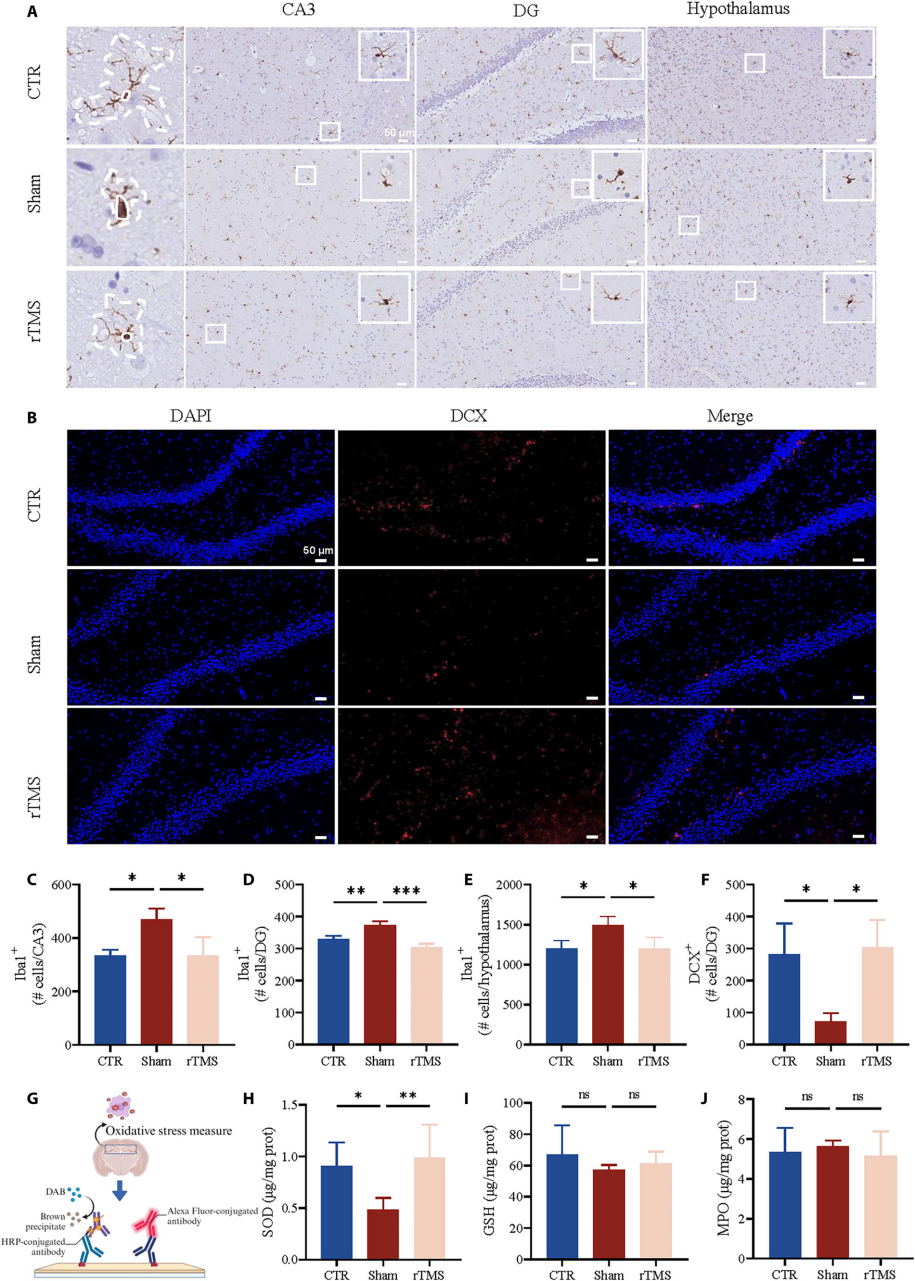
rTMS improved the HFD-induced neuroplasticity damage. (A) Representative microscope images of hippocampal and hypothalamus microglia in different groups. (B) Representative microscope images of DCX-positive neurons within the DG in different groups. (C to E) Quantification of Iba1^+^ cells in CA3, DG, and hypothalamus, respectively (*n* = 3). (F) Quantification of DCX^+^ neurons in DG in different groups (*n* = 3). (G) Schematic illustration of immunohistochemistry, immunofluorescence, and oxidative stress measure. HRP, horseradish peroxidase. (H to J) The expression of oxidative-stress-related indicators in the hippocampus in different groups (*n* = 5). Data are presented as means ± SD. ns *P* ≥ 0.05, *P< 0.05, **P < 0.01, and ***P < 0.001

**Fig. 3.**
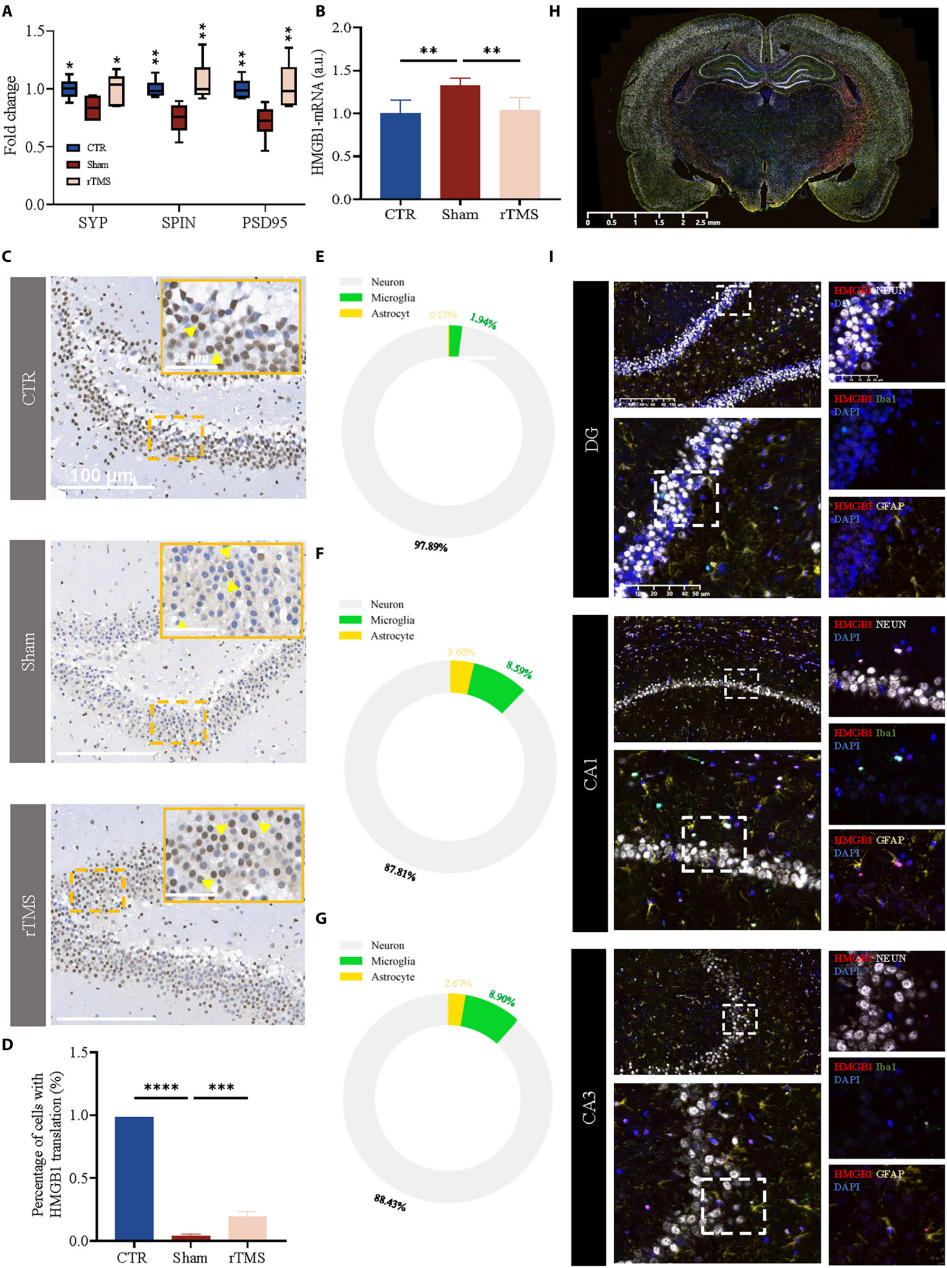
rTMS reduced the translation of HMGB1. (A) The mRNA expression of synapse-associated proteins including SPIN, SYP, and PSD95 in different groups. (B) The mRNA expression of HMGB1 in the hippocampus in different groups. a.u., arbitrary units. (C) Representative microscope images of HMGB1 in DG. (D) Quantification of the percentage of cells with HMGB1 translation in DG (*n* = 3). (E to G) Quantification of the proportion of HMGB1 expression in microglia, astrocytes, and neurons in DG, CA1, and CA3, respectively. (H) Representative images of immunofluorescence for Iba1, neuronal nucleus antigen (NeuN), glial fibrillary acidic protein (GFAP), and HMGB1. (I) Representative images of immunofluorescence for Iba1, NeuN, GFAP, and HMGB1 in DG, CA1, and CA3, respectively. Data are presented as means ± SD. ns *P* ≥ 0.05, *P< 0.05, **P < 0.01, ***P < 0.001, and ****P < 0.0001.

Alterations in the structure of hippocampal neurons are crucial in the onset and progression of cognitive decline [18]. Adult neurogenetic dysfunction in the hippocampus is thought to be the cause of spatial learning and memory dysfunction [19]. We first investigated whether an HFD affects hippocampal neurogenesis and whether rTMS can improve it. The HFD reduced the number of doublecortin^+^ (DCX^+^) in immature neurons in the DG, but rTMS reversed this change (Fig. 2B and F). Previous studies have shown reduced dendrite complexity in granular and pyramidal neurons and damage to synaptic ultrastructure in HFD-fed mice [20,21]. Changes in the expression of synapse-related proteins [spindlin 1 (SPIN); synaptophysin (SYP), and postsynaptic protein-95 (PSD95)] were confirmed through reverse transcription quantitative polymerase chain reaction (RT-qPCR) analysis. The findings indicated that rTMS led to a rise in the reduction of synapse-related proteins triggered by an HFD, as shown in Fig. 3A. These findings suggest that rTMS can partially ameliorate the negative morphological neuroplasticity effects of a long-term HFD on the hippocampus. Microglial activity and neuronal plasticity in neuropsychiatric disorders have been well established [22]. Activated microglia impair adult hippocampal neurogenesis and synaptic structures and lead to cognitive deficits [23]. By utilizing ionized calcium-binding adapter molecule 1 (Iba1) as a marker for microglia, it was noted that an HFD led to an increase in microglia in the cornu ammonis 3 (CA3) and DG areas of the hippocampus and hypothalamus. Conversely, rTMS notably decreased the cell count in these regions (see Fig. 2A and C to E). Microglial morphology is intricately linked to microglial function [22]. Microglia in the sham group showed higher levels of activation and were characterized by larger cell bodies with fewer branches.

### rTMS decreased hippocampal levels of some HFD-induced proinflammatory cytokines and increased hippocampal levels of vascular endothelial growth factor

Oxidative stress injury is also an important feature in HFD-fed rats, and, therefore, oxidative-stress-related indicators [superoxide dismutase (SOD), glutathione (GSH), and myeloperoxidase (MPO)] were assessed in the hippocampus. In comparison to the control group, SOD levels exhibited a significant decrease in the sham group but showed an increase in the rTMS group (Fig. 2H to J). HMGB1, a well-known protein that can trigger proinflammatory immune responses, is a typical damage-associated molecular pattern [24]. HMGB1 may be transferred to the cytoplasm to participate in the immune response under stress [25]. A significant increase in HMGB1 mRNA expression was observed in the sham group, reversed by the rTMS treatment (Fig. 3B). Similar results were obtained by immunohistochemistry, which showed that the proportion of HMGB1 transported to the cytoplasm in the DG was significantly increased in the sham group, which could be ameliorated by rTMS treatment (Fig. 3C and D). In addition, we found that HMGB1 was most highly expressed in neurons, followed by microglia, and less so in astrocytes, in each region of the hippocampus (Fig. 3E to I). HMGB1 mediates microglial activation, inducing excessive synaptic phagocytosis, neuronal dysfunction, and morphological abnormalities [26]. Blocking HMGB1 release with minocycline significantly improved cognitive deficits in depressive models [24]. rTMS may exert neuroprotective effects by counteracting the release of HMGB1, thereby ameliorating cognitive impairment.

**Fig. 4.**
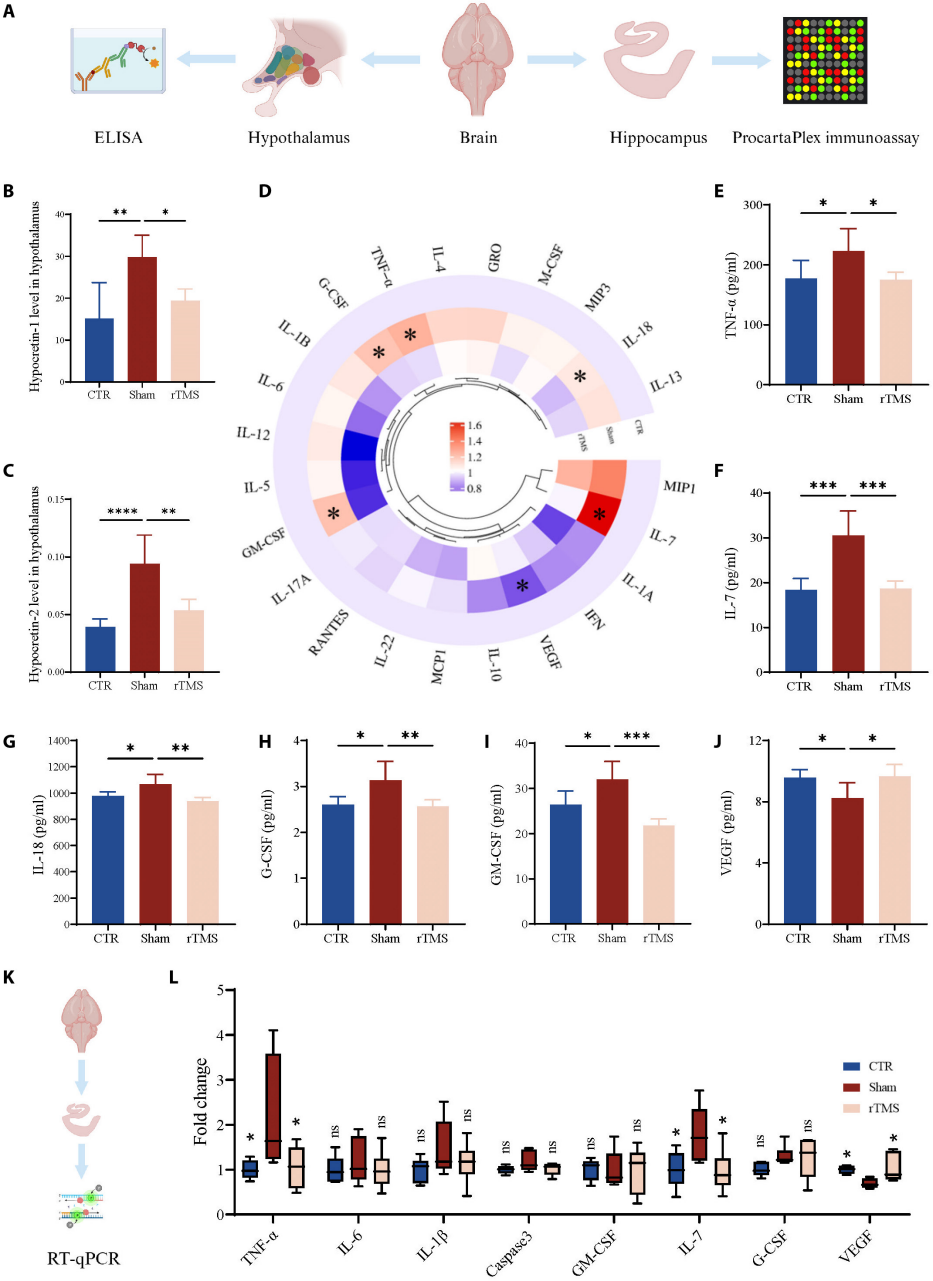
rTMS decreased proinflammatory cytokines and increased the VEGF levels. (A) Schematic illustration of ELISA and Luminex Liquid Suspension ChIP Assay. (B and C) Hypocretin-1 and hypocretin-2 levels in the hypothalamus in different groups (*n* = 6). (D) The expression of inflammation-related factors in the hippocampus in different groups (*n* = 5). (E) The expression of TNF-α in the hippocampus in different groups (*n* = 5). (F) The expression of IL-7 in the hippocampus in different groups (*n* = 5). (G) The expression of IL-18 in the hippocampus in different groups (*n* = 5). (H) The expression of G-CSF in the hippocampus in different groups (*n* = 5). (I) The expression of GM-CSF in the hippocampus in different groups (*n* = 5). (J) The expression of VEGF in the hippocampus in different groups (*n* = 5). (K) Schematic illustration of RT-qPCR. (L) The mRNA expression of inflammation-related factors (*n* = 6). Data are presented as means ± SD. ns *P* ≥ 0.05, *P< 0.05, **P < 0.01, ***P < 0.001, and ****P < 0.0001.

Levels of TNF-α, IL-7, IL-18, granulocyte colony-stimulating factor (G-CSF), and granulocyte-macrophage colony-stimulating factor (GM-CSF) in the hippocampus were lower in the rTMS group than in the sham group, as shown in Fig. 4D to I. Vascular endothelial growth factor (VEGF) levels in the hippocampus of the sham group notably decreased compared to the control group but increased following rTMS treatment (Fig. 4J). VEGF has been shown to maintain neuronal activity and promote neurogenesis, especially the number of DCX^+^ immature neurons [27,28]. We also used RT-qPCR to verify the expression levels of 3 groups of inflammation-related factors. The findings showed that TNF-α and IL-7 levels were higher in the sham group than in the CTR group but decreased following rTMS therapy (Fig. 4L).

### The link between hypocretin levels and inflammation

**Fig. 5.**
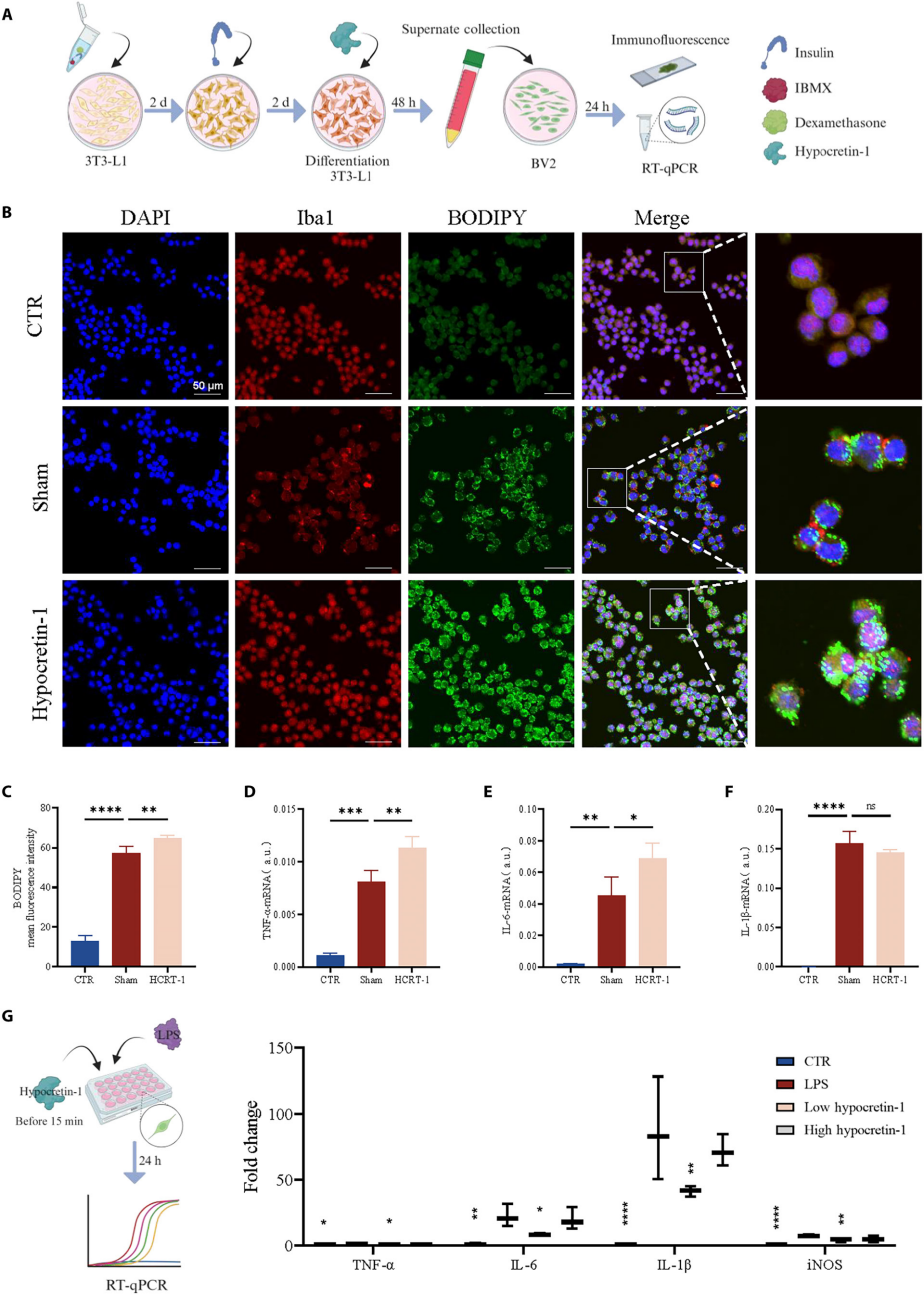
High hypocretin-1 levels may promote adipose accumulation and induce inflammation. (A) Schematic illustration of the effect of hypocretin-1 on lipid droplet accumulation and inflammatory factors expression in BV2. (B) Representative microscope images of BV2 stimulated by the conditional medium. Scale bar, 50 μm. (C) Quantification of BODIPY fluorescence intensity in different groups (*n* = 4). (D to F) The mRNA expression of TNF-α, IL-6, and IL-1β in different groups, respectively (*n* = 3). (G) Schematic illustration of the effect of different concentrations of hypocretin-1 on inflammatory factors expression in BV2. In addition, the mRNA expression of TNF-α, IL-6, IL-1β, and inducible nitric oxide synthase (iNOS) in different groups (*n* = 3). (ns) *P* ≥ 0.05, *P < 0.05, **P < 0.01, ***P < 0.001, and ****P < 0.0001.

Considering that hypocretin is involved in multiple functions such as inflammation, cognition, hypothalamic–pituitary–adrenal (HPA) axis, diet, etc., we studied the level of hypocretin in the hypothalamus. Enzyme-linked immunosorbent assay (ELISA) results revealed that an HFD induced an increase in hypocretin-1 and hypocretin-2, which decreased after rTMS treatment (Fig. 4B and C). Although previous studies have suggested that direct microinjection of hypocretin-1 does not directly affect the percentage of DCX^+^ cells in the DG [29], Forte et al. [30] reported that excessive hypocretin-1 may impair hippocampal neuron long-term potentiation and impair cognitive function. In addition, hypocretin has an anti-inflammatory effect [31], which seems to be contrary to our results. Therefore, we further used 3T3 and BV2 cell lines to simulate the effects of circulating hypocretin on high-fat environment and microglia (Fig. 5A). When the supernatant of 3T3 cells after differentiation was added to BV2 cells, lipid phagocytosis and the expression of inflammatory factors (TNF-α, IL-6, and IL-1β) were significantly increased in BV2 cells (Fig. 5B to F). However, the addition of hypocretin-1 to 3T3 cells further aggravated the inflammatory manifestations of BV2 and increased lipid phagocytosis (Fig. 5B to F). Lipopolysaccharide (LPS) has been shown to stimulate microglia to activate and secrete proinflammatory factors [32]. We used LPS to induce microglia to observe the direct effects of hypocretin-1 on them. We found that the anti-inflammatory effect was indeed better when the hypocretin-1 concentration was low, and the anti-inflammatory effect decreased as the hypocretin-1 concentration increased (Fig. 5G). We demonstrated that higher hypocretin levels by themselves do not have proinflammatory effects on BV2 cells. These results indicate that hypothalamic hypocretin-1 secretion increases after high lipid induction, and it may lose its anti-inflammatory effect.

### Gut microbiota dysbiosis induced by HFD was improved by rTMS

**Fig. 6.**
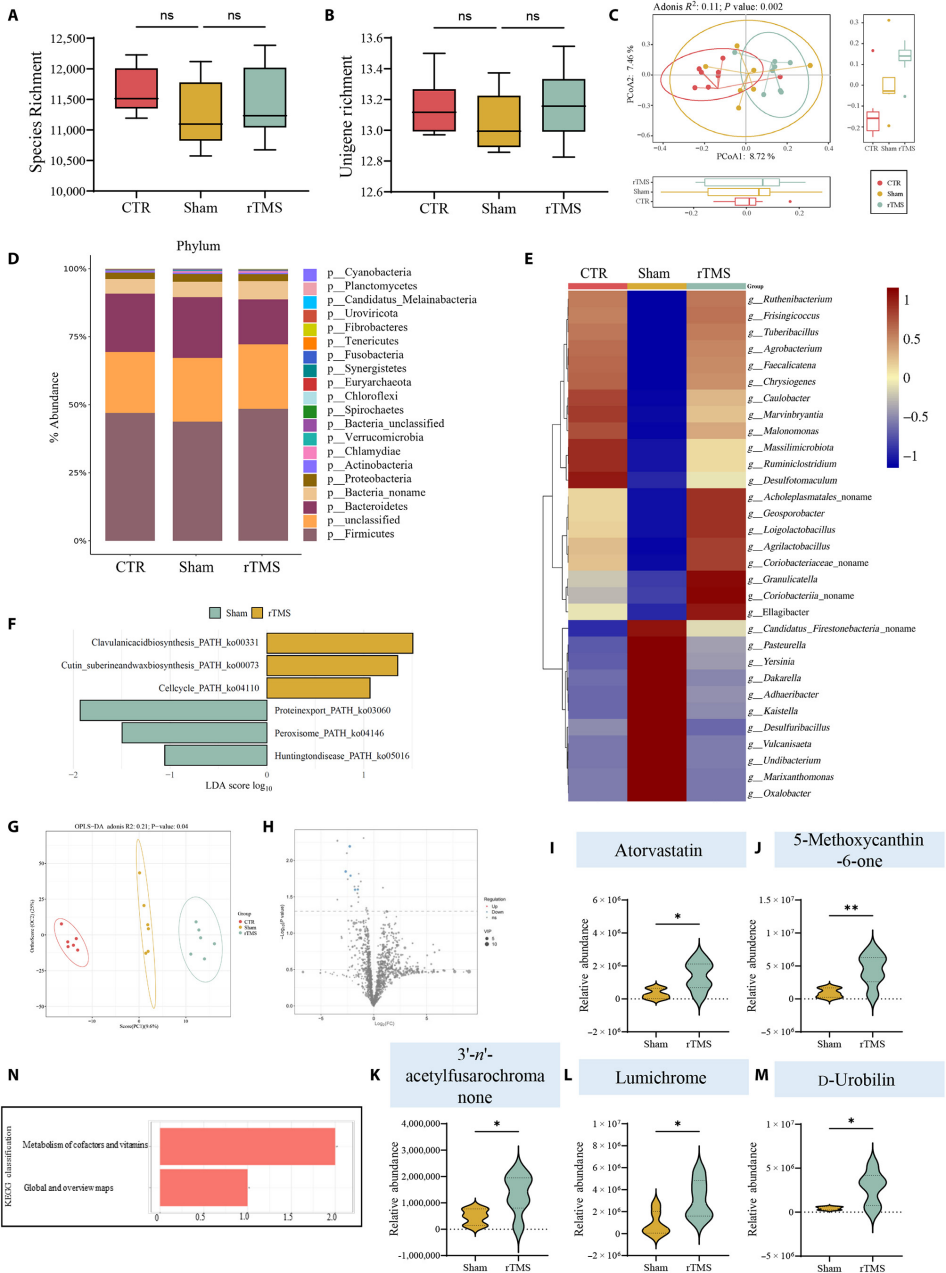
rTMS improved HFD-induced gut microbiota dysbiosis. (A and B) Changes in microbial richness at species and gene levels among different groups (*n* = 8 CTR, *n* = 7 sham, and *n* = 8 rTMS). (C) PCoA plots based on the metagenomic profiles of different groups. (D) The overall microbial characteristics at the phylum level in the different groups. (E) Heatmap of the top 30 different flora at the genus level in different groups. (F) The KEGG function predicted by PICRUSt identified potential functional interactions between the gut microbiome and the host in the sham and rTMS groups. (G) The intestinal metabolites in different groups based on orthogonal partial least square discriminant analysis. (H) The volcanic map showed the difference of metabolites between sham and rTMS groups. FC, fold change. (I to M) The relative abundance of metabolites in sham and rTMS groups, respectively. (N) The KEGG pathway analysis for sham and rTMS groups. Data are presented as means ± SD. ns *P* ≥ 0.05, *P< 0.05, and **P < 0.01.

The communication pathway between the brain and gut microbiota, known as the microbiota–gut–brain axis, has been associated with health and disease in recent research [33,34]. Current research has primarily focused on exploring the connection from the microbiome to the brain, with limited understanding of the opposite direction [35]. Hypocretin, as one of the most important chemical mediators in 2-way gut–brain dialog [36], showed significant changes before and after rTMS treatment. So, utilizing rTMS therapy for neuromodulation presents a valuable chance to enhance our comprehension of the communication pathway. Stool samples from 3 rat groups were subjected to shotgun metagenomic sequencing. Compared with CTR group and rTMS group, there was no significant difference in bacterial richness decline in sham group despite bacterial diversity (Fig. 6A and B). The utilization of principal coordinate analysis (PCoA) revealed significant differences in microbiota composition among the CTR, sham, and rTMS groups (Fig. 6C). Bar charts were used to present the overall microbial characteristics at the phylum level for the 3 categories, followed by hot spot maps illustrating the top 30 distinct flora at the genus level (Fig. 6D and E). In comparison to the other 2 groups, the sham group exhibited a higher abundance of potential harmful bacterial species such as *g-Pasteurella* and *Oxalobacter*. Conversely, protective bacteria including *g-Agrilactobacillus* and *g-Ruminiclostridium* were significantly reduced in the sham group. Linear discriminant analysis effect size (LEfSe) analysis demonstrated distinctive microorganisms across all levels for each of the 3 groups (Fig. S1A). In addition, Kyoto Encyclopedia of Genes and Genomes (KEGG) function prediction using PICRUSt revealed potential functional associations between gut microbiome and host in both sham and rTMS groups (Fig. 6F). Notably, compared to the sham group, pathways associated with clavulanic acid biosynthesis were significantly up-regulated in the rTMS group, while peroxisome-related pathways showed notable elevation in the sham group.

### rTMS improves hyperlipidemia by altering intestinal metabolites

**Fig. 7.**
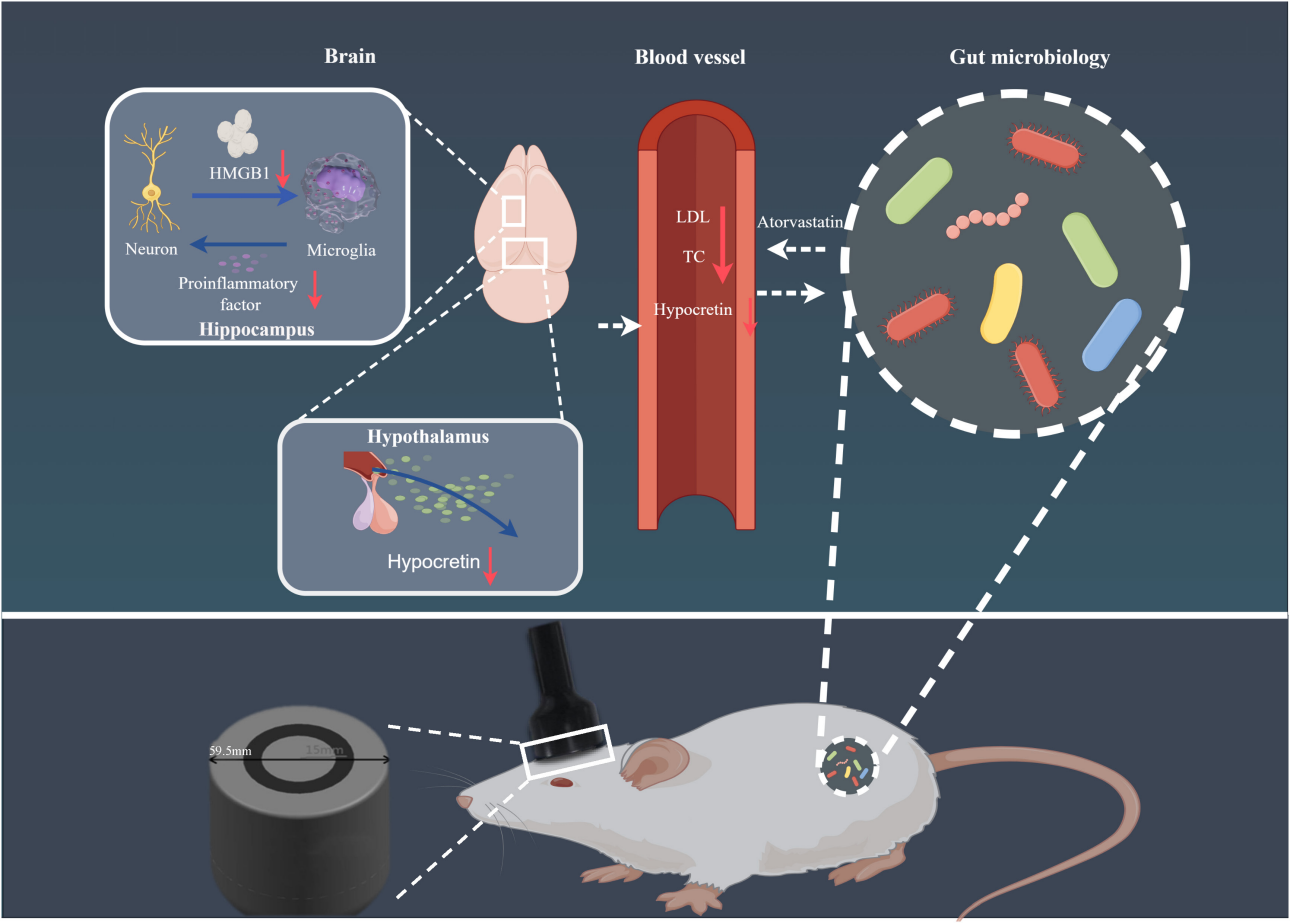
Schematic diagram of high-frequency rTMS in the treatment of HFD-induced obesity and cognitive impairment. rTMS reduces hippocampal neuron secretion of HMGB1 and down-regulates subsequent microglial activation and proinflammatory cytokine levels, thereby improving cognitive function in rats. rTMS can reduce the production of hypothalamic orexin and effectively restore blood lipids. At the same time, rTMS can maintain the balance of intestinal microbiome and intestinal metabolites in rats with HFDs. These changes may also be related to the improvement of blood lipids. Thus, rTMS may have beneficial effects on cognition through the brain–gut axis.

To uncover the metabolic changes induced by rTMS treatment, we performed metabolic analyses on stool samples from 3 groups of rats. The intestinal metabolites of the 3 groups of rats were significantly different by orthogonal projections to latent structure-discriminant analysis (OPLS-DA) (Fig. 6G). The volcanic map showed the difference of metabolites in sham and rTMS groups, including atorvastatin, 5-methoxycanthin-6-one, 3′-*n*′-acetylfusarochromanone, lumichrome, and d-urobilin (Fig. 6H to M). Of these pathways, metabolism of cofactors and vitamins were the most varied in rats treated with rTMS (Fig. 7N). Notably, atorvastatin has been shown to significantly reduce blood cholesterol, LDL cholesterol levels, and serum TG levels [37].

A comprehensive analysis was performed to identify potential associations between changes in gut microbiota and metabolites in rats treated with rTMS. We found that the enrichment of *Agrilactobacillus*, *Extibacter*, and *Lachnoclostridium* was positively correlated with atorvastatin (Fig. S1B) and negatively correlated with hypocretin-1 and hypocretin-2 concentrations. Furthermore, the enrichment of *Adhaeribacter* and microvirus showed a negative association with atorvastatin levels but a positive association with hypocretin-1 and hypocretin-2 concentrations as well as HMGB1 expression. These results suggest that there may be a potential microbiota–gut–brain link and that rTMS treatment does improve the rat gut microbiota and its associated metabolites.

## Discussion

In this study, we found that long-term HFDs reduced cognitive function in rats and that high-frequency rTMS improved cognitive deficits in rats fed an HFD and improved neurogenesis and hippocampal neuroinflammation. In addition, we report that high-frequency rTMS can effectively restore changes in the gut microbiome of HFD rats; in particular, we find that rTMS treatment reduces the abundance of microvirus, which has not been reported in previous studies, and we find that intestinal metabolites may be associated with changes in blood lipids. Overall, these data provide robust support for the potential therapeutic efficacy of rTMS in addressing cognitive impairment induced by an HFD along the gut–brain axis.

Clinical studies have confirmed that rTMS can reduce weight and suggest that decreased food intake due to decreased appetite may be the mechanism of rTMS weight loss [38]. Several studies have reported that compared with the sham operation group, the appetite and hunger of the rTMS group are reduced, suggesting that rTMS can increase the sense of satiety [39,40] . Consistent with clinical studies, our results show that rTMS reduces food cravings in rats. Our results further reveal that this may be due to the down-regulation of hypothalamic hypocretin levels by rTMS. However, studies have shown that hypocretin has anti-inflammatory effects [41], which seems to contradict our findings in obese rats. In obese rats induced by HFD, microglia increased significantly in hippocampus, the secretion of proinflammatory factors increased, and the level of hypocretin in hypothalamus increased as well. In subsequent cell tests, we found that when the level of hypocretin-1 increased, the anti-inflammatory effect did not increase but decreased. Meanwhile, when hypocretin-1 acts on fat cells, fat cells may secrete factors that promote neuroinflammation, thus exacerbating the production of neuroinflammation. These results explain the coexistence of high hypocretin levels and neuroinflammation in rats induced by an HFD.

Exposure of immune cells to microbial-associated molecular patterns, pathogen-associated molecular patterns, and endogenous inflammatory mediators leads to the activation of immune cells and the modulation of inflammatory responses by HMGB1 [42]. Microglia represent the predominant population of innate immune cells in the brain that actively release HMGB1 [25]. HMGB1 possesses the capability to bind to surface receptors on microglia, thereby initiating inflammatory pathways and augmenting microglial activation [26]. The translocation of HMGB1 between the nucleus and cytoplasm is a crucial process for its active release [43]. The release of HMGB1 has been associated with cognitive impairments in diabetes-related dementia, depression, and Alzheimer’s disease [44]. Under pathological conditions, HMGB1 can enhance the phagocytosis of microglia, leading to overexpression of inflammatory mediators, abnormal synaptic pruning, neuronal dysfunction, and cognitive impairment [26]. rTMS reduces the translocation of HMGB1 in microglia and neurons, reduces microglial activation and proinflammatory cytokine secretion, increases VEGF levels, restores hippocampal neurogenesis, and thus improves cognitive dysfunction induced by HFD.

Two-way gut–brain axis communication has been widely accepted [45]. The brain exerts direct regulatory control over the gastrointestinal nervous systems via the parasympathetic and sympathetic branches of the autonomic nervous system, as well as through the HPA axis. In addition, it can indirectly modulate the gut microbiota by modifying the microenvironment within the gut [46]. Therefore, neuromodulation techniques may affect the composition and function of the gut microbiota. Previous studies have extensively reported the pathogenesis of intestinal flora involved in obesity or cognitive impairment, including the relationship between characteristic intestinal flora, intestinal flora diversity, and the severity of insulin resistance in obese patients [47]. We found no significant difference in the complexity of the gut microbiota among the 3 groups. We found that probiotics such as *Ruminiclostridium*, *Loigolactobacillus*, *Agrilactobaccillus*, and *Ellagibacter* significantly decreased in the obese group, while harmful bacteria such as yersinia and *Adhaeribacter* significantly increased. These are reversed by rTMS. Notably, we found that rTMS reduced microvirus levels. The shift from normal foods to an HFD led to a significant decrease in phages from the Siphoviridae family, accompanied by an increase in Microviridae [48]. Another study also showed that those with high levels of Microviridae did worse on cognitive tests and that an HFD led to increased levels of Microviridae and their levels were positively correlated with fat mass. Mice transplanted with high levels of Microviridae also tended to perform worse on cognitive tests [49]. Besides the imbalance of the gut microbiota, obesity may also be influenced by the small-molecule metabolites generated by dietary foods and symbiotic bacteria. Gut microbiome related metabolites may ameliorate or exacerbate the development of intestinal obesity [49]. Thus, we reveal the role of intestinal metabolite alterations in rTMS treatment of HFD. Therefore, our findings suggest that even just 3 weeks of rTMS treatment promotes beneficial changes in the microbiome composition of obese rats induced by HFD.

Of course, there are some limitations to this study. First, we demonstrated that rTMS can improve the cognitive dysfunction and intestinal microecology disturbance induced by HFD in rats, without further verifying the direct causal relationship between intestinal microecology and brain. Second, we did not further explore the optimal rTMS parameters. This study mainly confirmed whether rTMS is effective for cognitive dysfunction caused by HFD, but it is an interesting research direction to explore the optimal rTMS parameters in the future. Finally, because of the small size of the rat brain, the stimulation of the coil cannot be accurately targeted to a specific brain region. In fact, most studies of TMS mechanisms in rodents have been based on whole/half-brain stimulation, and the lack of a rodent-specific high-focus TMS coil has also greatly limited the translation of treatment options for human TMS into animal models to explore the mechanisms. In the future, new magnetic materials might be used to solve this problem [50–52], improving the spatial focusing of TMS coils.

## Materials and Methods

### Animals and treatments

Animal experiments were carried out following the guidelines of the Ethics Committee at the First Affiliated Hospital of Zhejiang University School of Medicine. Attempts were undertaken to alleviate the distress of the rodents and decrease the quantity of rodents utilized. Each animal was kept in a separate ventilated cage in a controlled environment with a temperature of 22 ± 2 °C, a 12-h light/dark cycle, and access to food and water at all times. Eight-week-old male SD rats were randomly divided into CTR, sham, and rTMS groups. The CTR group received regular diet, while both sham and rTMS groups received HFD for 12 weeks. rTMS was administered using a magnetic stimulator (Jiangxi Brain Modulation Function), lasted for 21 d. In the rTMS group, the left dorsolateral prefrontal lobe’s skull was positioned at the center of a compact coil and exposed to 1,200 pulses, comprising 12 series of 100 pulses administered at a frequency of 10 Hz with an intensity of 1.26 T/d. Sham group was treated similarly to the rTMS group and listened to a ticking sound at 10 Hz but was not stimulated.

The day after the end of the behavioral studies, feces were collected for metagenomic and metabolomic studies. The rats were anesthetized with 4% isoflurane and euthanized by guillotine. Plasma was obtained by centrifuging blood samples at 3,000 rpm for a duration of 10 min. Hippocampal and hypothalamic tissues were also separated on ice. The plasma and brain tissue were kept at −80 °C until they were needed. Several rats were sedated with pentobarbital injected into the peritoneum and then underwent transcardiac perfusion with phosphate-buffered saline (PBS) and 4% paraformaldehyde before their brain tissue was extracted and preserved for paraffin sectioning.

### Behavior tests

#### Open field test

OFT is an experimental method to test the spontaneous activity behavior and exploratory behavior of rats for assessing anxiety-like behavior. Briefly, the rats were placed in a black open box (100 cm × 100 cm × 100 cm), with an area of 25 cm × 25 cm near the center point considered to be the central zone, and the total distance within 5 min was recorded. Odor was eliminated by wiping with 70% alcohol before the second rat was tested.

#### Elevated plus maze

The EPM is an experimental method used to assess anxiety responses in rodents. Positioned around 70 cm above the floor, the structure featured 2 exposed arms and 2 enclosed arms. The rats were positioned in the middle section and examined for 5 min with their snouts directed toward one of the enclosed corridors. The time spent in the open arms was recorded, which was negatively correlated with the anxiety of the rats.

#### Novel object recognition test

NOR reflects the cognitive ability of rats by measuring their exploration of old and new objects, divided into an adaptation phase and a test phase. During the adjustment period, 2 indistinguishable items (without scent or pressure) were positioned in the exposed area, and the rats were introduced with their backs turned toward the items and allowed to freely investigate for 5 min. One hour after the rats had adjusted, they were moved into the testing phase. During this phase, one object was swapped out for another, and the rats were positioned with their backs facing the object at an equal distance to investigate for 5 min. The number and the duration of explorations of the old and new objects were recorded with the criterion that the mouth or nose touched the object or came within about 2 to 3 cm of the object.

#### Y-maze test

The Y-maze consisted of 3 arms and was divided into training and testing periods. Throughout the training session, a partition obstructed the new arm, while the rat was placed in the starting arm to roam freely for 5 min. While the partition of the new arm was removed in the test period, and the rat was put in by the starting arm to move freely in all 3 arms for 5 min. The residence time of the rat in each arm was recorded.

### Biochemical parameter analysis

Levels of LDL, HDL, TC, and TG were assessed with the corresponding test kits from Nanjing Jiancheng Bioengineering Institute, following the provided guidelines.

### Immunohistofluorescence and immunohistochemistry

Paraffin sections were treated with a series of ethanol solutions (100%, 100%, 96%, 90%, 80%, 70%, 60%, and 50%), followed by antigen retrieval using EDTA buffer (pH 6.0), cooling down, rinsing 3 times with tris-buffered saline (TBS), and blocking with 3% bovine serum albumin for 30 min at ambient temperature. Following a gentle shake to remove the blocking solution, the sections were then exposed to the primary antibodies (DCX, Abcam; Iba1, Abcam) overnight at 4 °C.

In immunohistofluorescence, the sections were washed 3 times with TBS, followed by incubation with the suitable secondary antibody at room temperature for 1 h. Subsequently, 4′,6-diamidino-2-phenylindole (DAPI) stain was added drop by drop and left to incubate for 10 min at room temperature in the dark. After another 3 washes with TBS, the sections were sealed using an antifluorescence quenching sealer. Sections were viewed using a Nikon inverted fluorescence microscope, and photographs were taken.

Immunohistochemistry involved washing sections 3 times with tris-buffered saline with Tween 20 (TBST), followed by incubation with the suitable secondary antibody at 37 °C for 45 min. After another round of TBST washing, the sections were treated with freshly made diaminobenzidine. The reaction was halted in tap water, and the nuclei were stained with hematoxylin for 1 min. Subsequently, the sections were washed and differentiated in hydrochloric acid alcohol for 1 to 2 s before being blocked with neutral resin.

### Oxidative stress measures

Levels of SOD, GSH, and MPO in the hippocampus were measured with a commercially available kit (Solarbio, BC5165 and BC1175; Nanjing Jiancheng Bioengineering Institute, A044-1-1) according to the instructions provided by the manufacturer.

#### Enzyme linked immunosorbent assay

Levels of hypocretin-1 and hypocretin-2 in the hypothalamus were measured as per the instructions provided by the manufacturer. Briefly, hypothalamic tissues were crushed using mechanical means in a mixture of 1 part of PBS to 9 parts of water (1:9, v/v) while keeping cold and then spun at 12,000 rpm for 15 min, and the liquid above the sediment was gathered for analysis.

#### Luminex liquid suspension chromatin immunoprecipitation assay

Luminex Liquid Suspension Chromatin Immunoprecipitation (ChIP) Assay was performed at Wayen Biotechnology (Shanghai, China). In summary, the levels of each factor in the hippocampus were measured according to the instructions of the Bio-Plex system.

### Cell lines and conditions

BV2 and 3T3-L1 cells obtained from Zhejiang Baidi Biotechnology Co. Ltd. and Procell Life Science & Technology Company were cultured in minimum essential medium and Dulbecco’s modified Eagle’s medium (BDBio, China, L100-500) medium with 10% fetal bovine serum (BDBio, China, F801-500) and 1% penicillin and streptomycin at 37 °C and 5% CO_2_, respectively. To distinguish, 3T3-L1 cells were seeded in plates for 2 d, followed by a switch to full medium with 0.5 mmol of 3-isobutyl-1-methylxanthine, 1 μmol of dexamethasone, and insulin (10 μg/ml) for 2 d and then a switch to full medium with insulin (10 μg/ml). 3T3-L1 cells that were treated differently were subjected to the following conditions for a period of 48 h: (i) the same quantity of PBS and (ii) 200 nM hypocretin-1. The medium was gathered individually, and the liquid above was kept following centrifugation at 12,000 rpm for 5 min.

BV2 cells were exposed to the following conditions for 24 h, respectively: (i) an equal amount of PBS (CTR group), (ii) supernatant of differentiation 3T3-L1 medium (sham group), and (iii) supernatant of differentiation 3T3-L1 plus hypocretin-1 medium (hypocretin-1 group).

BV2 cells were exposed to the following conditions for 24 h, respectively: (i) an equal amount of PBS (CTR group), (ii) LPS (1 μg/ml; Sigma-Aldrich, LPS group), (iii) LPS (1 μg/ml) plus hypocretin-1 [100 nM; MedChemExpress (MCE), 15 min before LPS, low-hypocretin-1 group], and (iv) LPS (1 μg/ml) plus hypocretin-1 (500 nM; MCE, 15 min before LPS, high-hypocretin-1 group).

### Immunocytochemistry

After being inoculated on a circular coverslip, the cells were treated with 4% paraformaldehyde for 15 min at ambient temperature and then rinsed 3 times with PBS. The barrier was ruptured and obstructed using 0.1% Triton X-100 in 2% bovine serum albumin at 37 °C for a duration of 30 min. Primary antibody (Iba1, Abcam; 1:500) was incubated at 4 °C overnight, followed by secondary antibody (CoraLite647-conjugated goat anti-rabbit, Proteintech; 1:500) incubation for 1 h at room temperature, shielded from light. Next, lipid droplets were stained using BODIPY (10 μM; MCE) for 30 min at room temperature, protected from light. The nuclei of the cells were stained with DAPI for 10 min. After washing 3 times with PBS, the samples were sealed with an antifluorescence quenching sealer. Specific fluorescence signals were examined using a confocal microscope (FV3000).

### Real-time quantitative polymerase chain reaction

Briefly, we isolated total RNA from hippocampal tissue and BV2 cells using the Fast Pure Total RNA Isolation Kit (Vazyme, #RC112-01), then converted it to cDNA (Vazyme, R333-01) before conducting RT-qPCR with the following steps: 95 °C for 2 min and 40 cycles of 95 °C for 15 s, 60 °C for 30 s, and 72 °C for 30 s. The data were analyzed using the 2^−ΔΔCt^ method, and the mRNA levels of the target gene were standardized against β-actin. Tables 1 and 2 display the primer sequences.

**Table 1.** The list of primers used in RT-qPCR for BV2

Gene	Primer sequences (Forward)	Primer sequences (Reverse)
Tnf	GGCAGTTAGGCATGGGATGA	TCCACTTGGTGGTTTGCTGA
Il6	TCCTACCCCAATTTCCAATGCT	CGCACTAGGTTTGCCGAGTA
Il1b	TGCCACCTTTTGACAGTGATG	TGATGTGCTGCTGCGAGATT
Actb	TGCGGGATGGTCAGTTAAGAG	CCTTCTGACCCATTCCCACC

Tnf, tumor necrosis factor; Il6, interleukin 6; Il1b, interleukin 1 beta; Actb, actin beta

**Table 2.** The list of primers used in RT-qPCR for rat

Gene	Primer sequences (Forward)	Primer sequences (Reverse)
Tnf	CATCCGTTCTCTACCCAGCC	AATTCTGAGCCCGGAGTTGG
Il6	CACTTCACAAGTCGGAGGCT	TCTGACAGTGCATCATCGCT
Il1	TGGCAACTGTCCCTGAACTC	CATCTGGACAGCCCAAGTCA
Hmgb1	GGCTTTTCCCCATTAACAACACT	ATTTTGGAAGCTGGTCCCCT
Casp3	GAGCTTGGAACGCGAAGAAA	GAGTCCATCGACTTGCTTCCA
Csf2	CCCCGGAAACTGACTGTGAA	CCTCATTTCTGGACCGGCTT
Il7	AACCATGCATCGGTGAACCA	TGTGACTCTCGTTTTCAAGAGC
Csf3	GTTGAACACCACTGTTGCCC	CCACTCGGTGGACAACATCA
Vegf	TGACCACATTCACTGTGAGCCT	ACGCGAGTCTGTGTTTTTGC
Syp	TCCTGTACCCTCTGCTGTGT	GCACAGGAAAGTAGGGGGTC
Spin1	TAGATGTGCGGTGCGGATAC	CCATCCAGGTGTCGCCTTTA
Dlg4	TACCGCTACCAAGATGAAGACA	GTTCCATTCACCTGCAACTCA
Actb	GCGAGTACAACCTTCTTGCAGC	TGCCGGAGCCGTTGTCG

Tnf, tumor necrosis factor; Il6, interleukin 6; Il1, interleukin 1; Hmgb1, high mobility group box 1; Casp3, caspase 3; Csf2, colony stimulating factor 2, also Gm-csf; Il7, interleukin 7; Csf3, colony stimulating factor 3, also Gcsf; VEGF, vascular endothelial growth factor; Syp, synaptophysin; Spin1, spindlin 1; Dlg4, discs large MAGUK scaffold protein 4, also PSD95; Actb, actin, beta.

### Shotgun metagenomic sequencing and taxonomic annotation

DNA from rat feces was isolated with the TIANamp Stool DNA kit from Tiangen Biotech and assessed for purity and integrity. Rat fecal DNA was subjected to shotgun metagenomic sequencing using an Illumina NovaSeq 6000 platform as in our previous study [53].

### Metabolomics analysis of fecal samples

Metabolite extraction from fecal samples and metabolomics data processing and analysis were performed as described in our previous study [54].

### Statistical analysis

GraphPad Prism software was utilized for conducting statistical analyses. The data were displayed as the average ± the SD for variables that followed a normal distribution. The comparison of the 2 groups was assessed using an unpaired Student’s *t* test. Differences among the experimental groups were determined by one-way analysis of variance (ANOVA), followed by Dunnett’s test for post hoc comparisons. *P* < 0.05 signified a significant statistical discrepancy.

## Data Availability

All data relevant to the study are included in the article or uploaded as supplementary information. Additional data relevant to the article are available from the corresponding author upon request.
