# Supplementary material for: Repetitive Transcranial Magnetic Stimulation (rTMS) Improves Cognitive Impairment and Intestinal Microecological Dysfunction Induced by High-Fat Diet in Rats
Source: Research (Wash D C). 2024 May 31;7:0384. doi: 10.34133/research.0384 (PMC11140411; doi:10.34133/research.0384)

**
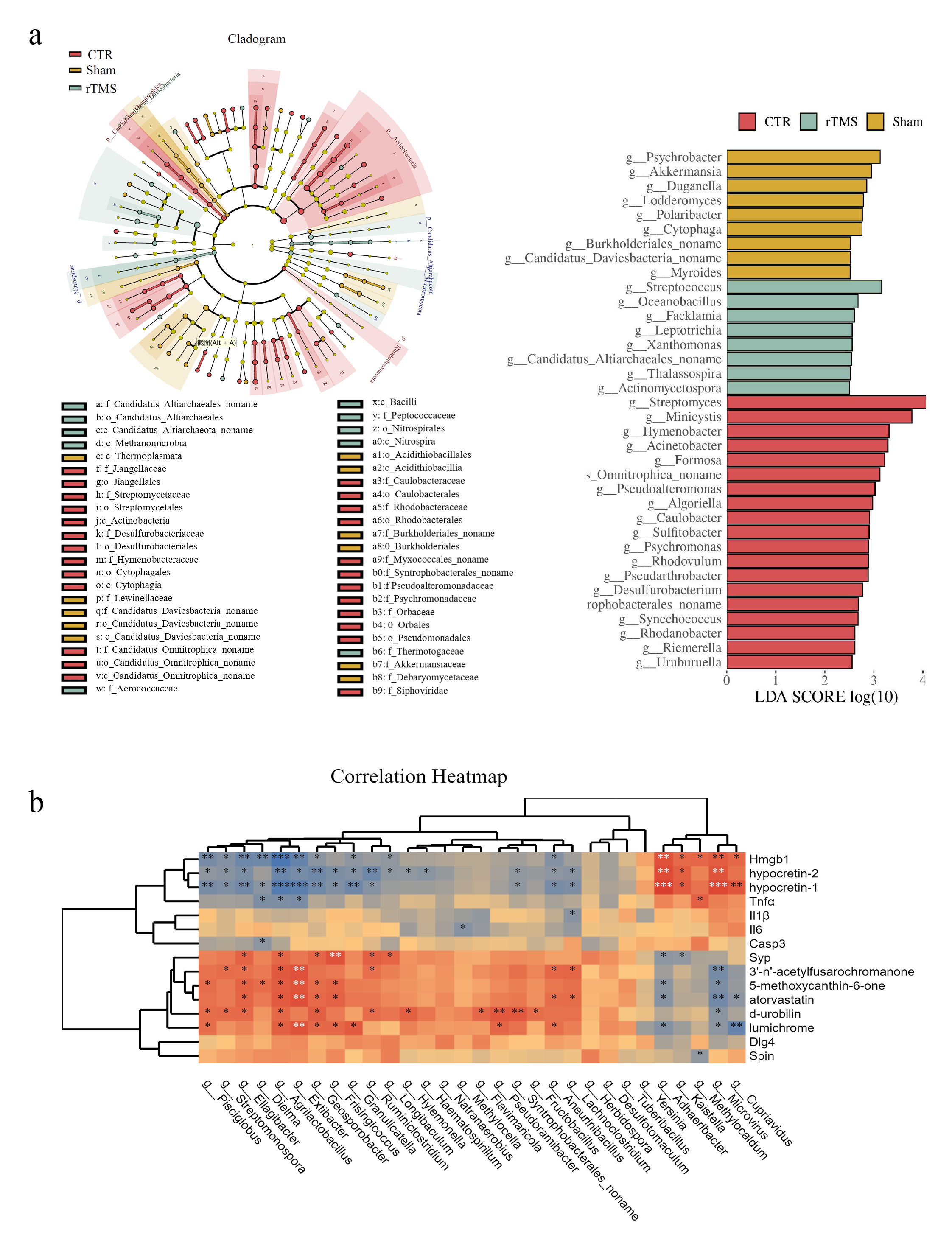
**

**Supplementary Figure 1. a** Differential abundance microbiota taxonomic cladogram in different groups at all levels obtained from LEfSe analysis, and differential abundance microbiota taxonomic cladogram at the genus level in different groups. **b** The correlation heatmap of gut microbiota and metabolites. Not significant (ns) *p* ≥ 0.05, **p*< 0.05, ***p* < 0.01 and ****p* < 0.001.


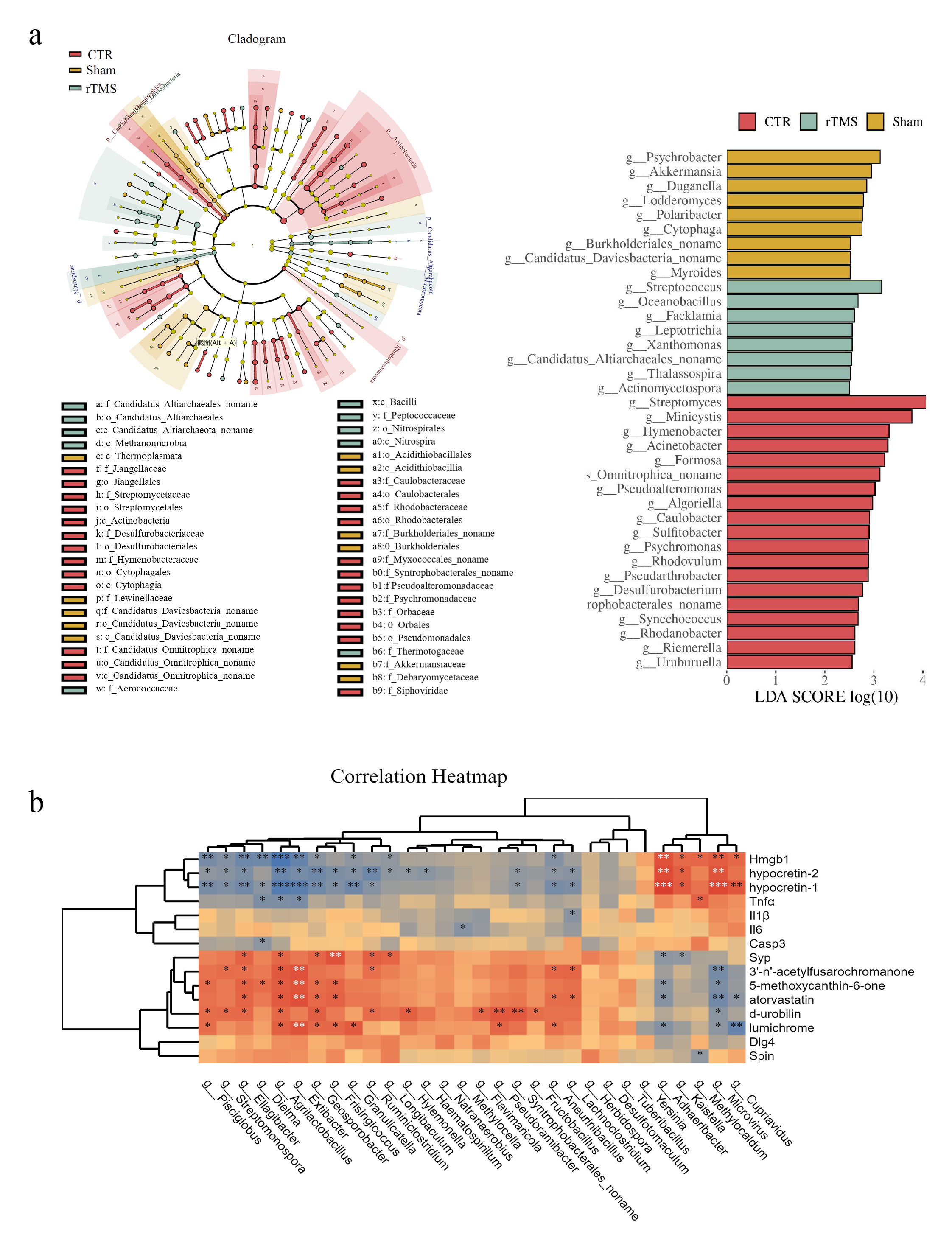

Supplement: Supplementary 1 — Fig. S1 [file research.0384.f1.docx]
